# Transporter oligomerisation: roles in structure and function

**DOI:** 10.1042/BST20180316

**Published:** 2018-12-21

**Authors:** Cristina Cecchetti, Euan Pyle, Bernadette Byrne

**Affiliations:** Department of Life Sciences, Imperial College London, London SW7 2AZ, U.K.

**Keywords:** function, membrane lipid, oligomerisation, protein structure, transporter

## Abstract

Oligomerisation is a key feature of integral membrane transporters with roles in structure, function and stability. In this review, we cover some very recent advances in our understanding of how oligomerisation affects these key transporter features, with emphasis on a few groups of transporters, including the nucleobase ascorbate transporters, neurotransmitter sodium symporters and major facilitator superfamily members.

## Introduction

Integral membrane transporters play key roles in the effective movement of specific molecules across biological membranes. Understanding of how these important proteins function is increasing rapidly due to the determination of high-resolution structures, biochemical and functional analysis, and the application of emerging technologies including advanced mass spectrometry techniques. Many transporters form oligomers that are important for effective trafficking to the membrane, function and/or regulation of function. This review covers the very recent advances that have been made in (1) understanding how these oligomers are stabilised in the membrane with emphasis on the role of lipids and (2) understanding of the role of transporter oligomers in a range of different families.

## Lipids and transporter oligomerisation

The role of membrane lipids in the formation of oligomers is an emerging feature of transporters. Membrane lipids form the native environment of integral membrane transporters. Some membrane lipids form a so-called annulus or ring around individual protein molecules. These lipids stabilise the protein through formation of non-specific H-bonds with the lipid head groups and through hydrophobic matching where the length of the hydrophobic regions of the protein is matched by that of the tail regions of the lipids [1]. Membrane lipids can also form specific, high-affinity interactions with membrane proteins. These interactions often play key roles in membrane protein structure and function.

A major step forward in the study of these interactions has been the advent of advanced mass spectrometry (MS) methods, such as native MS, allowing analysis of membrane protein/lipid complexes in detergent-based solution [2,3], nanodiscs [4] or amphipols [5]. These approaches permit examination of protein: lipid interactions that are often not detectable even in high-resolution structures. The preparation of proteins for analysis in detergent-based solution involves the removal of all but the most tightly associated lipids, and thus only those lipids most likely to form specific interactions remain bound. Proteins can also be reconstituted in nanodiscs containing similar lipids to the cell membrane to replicate similar protein–lipid interactions to those found *in vivo* although the protein is prepared initially by detergent extraction and purification [4].

## Nucleobase ascorbate transporters

**Figure 1.**
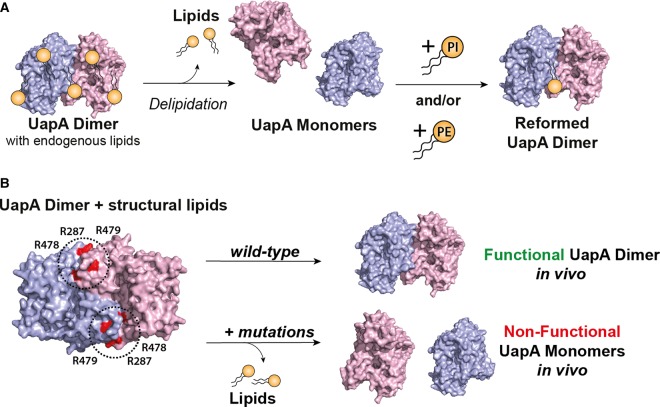
Functional UapA dimer formation is dependent on membrane lipids. (**A**) UapA in detergent-based solution exists normally as a dimer in complex with membrane lipids as determined by native MS. Delipidation results in dissociation of the UapA dimers into the monomeric form. Dimers can be recovered by the addition of PI and PE both alone and in combination. (**B**) A potential lipid-binding site, comprising R287, R478 and R479, at the interface between the two protomers, was identified by molecular dynamics (MD) simulations. Mutation of all three Arg residues to Ala results in a loss of UapA transport function in the native host, *Aspergillus nidulans*. This triple mutant isolates almost exclusively in the monomeric form.

Native MS analysis showed that a conformationally locked mutant (G411V_Δ1-11_) of the uric acid/xanthine transporter, UapA, a nucleobase ascorbate transporter from *Aspergillus nidulans*, isolates as a dimer [6], confirming earlier structural and biochemical studies [7,8]. Native MS also revealed the presence of UapA–lipid complexes that are retained even after protein purification in detergent. Lipidomics analysis identified that the phospholipids, phosphatidylinositol (PI), phosphatidylethanolamine (PE) and phosphatidylcholine (PC), co-purify with UapA. Removal of these lipids causes dissociation of the dimer into the monomeric form of the protein, while the addition of PI and PE alone, or in combination, to the delipidated protein results in dimer reformation (Figure 1). Molecular dynamics simulations using the inward facing structure of the UapA–G411V_Δ1-11_ [8] predicted a lipid-binding site at the dimer interface comprising three Arg residues. Subsequent mutagenesis of the residues comprising the lipid-binding site retained trafficking to the membrane and substrate-binding ability but lost xanthine transport function in the native host [6]. Native MS revealed that a mutant lacking the lipid-binding site purifies as a monomer and does not retain protein–lipid interactions (Figure 1). UapA is known to function only as a dimer; hence, these data strongly indicate that the lipids are critical for dimer formation and thus transport activity.

That UapA dimer formation is highly dependent on lipid binding was somewhat surprising given the large dimer interface (6000 Å^2^) formed by the two protomers, and precisely why specific, high-affinity lipid binding is required at this site remains unclear. It is thought that UapA functions via an elevator model [9] whereby the core, or substrate-binding domain, undergoes a substantial conformational change moving against the relatively static gate, or dimerisation domain, in order to transport cargo from one side of the membrane to the other. Lipid binding might be critical for stabilising the dimer interface and thus the whole dimeric molecule during this large conformational change of the core domain. However, the lipids remain critical for dimer formation in the conformationally locked, inward facing UapA–G411V_Δ1-11_ construct, so it seems more likely that these lipids are key for general stability and maintenance of the UapA dimer.

Similar approaches have demonstrated that lipid binding is key in stabilising a range of other oligomeric transporters. An analysis of many transporters ranked the strength of interaction based on buried surface area of the oligomer, giving a measure of so-called oligomeric stability [10]. The data indicated that oligomerisation showed greater lipid dependence in proteins with smaller interaction surfaces, for example the bacterial leucine transporter, LeuT, than those with larger interaction surfaces, such as the sodium proton antiporter, NapA, from *Thermus thermophilus* [10]. While this is a logical finding, clearly the data from UapA indicate that lipid stabilisation of transporter oligomerisation is not always a function of the size of the interaction interface.

What is becoming increasingly clear is that many functional transporter units are complexes of protein and membrane lipid.

A structure of the uracil transporter, UraA, from *Escherichia coli* was solved several years ago in the inward facing conformation and reported as a monomer [11]. More recently, an additional structure of UraA in the occluded state was solved of the protein as a dimer [12]. As with UapA, the UraA dimer interface is mediated exclusively through the gate domain. In addition, UraA mutants trapped in a monomeric state exhibit no transport activity although they bind substrate with WT affinity [12]. Therefore, the UraA dimer is almost certainly the physiological form of the protein. The co-expression of functional and non-functional transporter forms was used as a tool to further explore the role of the dimer in function [8,12]. In the case of UapA, non-functional mutants exert a dominant negative effect on an associated WT transporter resulting in a completely non-functional dimer [8]. Intriguingly, in the case of UraA, dimers of WT + a non-functional mutant expressed as a fusion protein exhibit transport activity very similar to WT [12]. It is possible that the very close interaction between the UapA monomers means that there has to be a co-ordinated action between the individual protomers in order to drive the conformational rearrangements associated with the transport, as has been suggested for GLUT1 [13] (see the section ‘Major Facilitator Superfamily Transporters’ below), while in UraA the slightly looser association may allow independent activity of each protomer. A high-speed atomic force microscopy study has demonstrated that the individual protomers in the trimer of sodium/aspartate symporter, Glt_PH_ from *Pyrococcus horikoshii*, reconstituted into lipid vesicles lack cooperativity [14]. In the case of both UraA and Glt_PH_, it is possible that oligomerisation simply plays a role in membrane stability of the transporters.

The NAT proteins are structurally related to the SLC4 transporter family which includes anion exchanger 1 (AE1) from humans [15]. Three structures of SLC4 family members have also been recently described as dimers [16–18]. Like the NAT proteins, the SLC4 transporters are suggested to function via an elevator mechanism. In the case of all these proteins, there is no evidence as yet that the oligomeric state is functionally relevant beyond the stability conferred by the protein : protein interaction.

## The neurotransmitter–sodium symporter family of transporters

Oligomerisation is a feature of the neurotransmitter–sodium symporter family of transporters but is not a requirement for native uptake activity [19]. Recent research demonstrated a direct interaction between the serotonin transporter (SERT) and the membrane lipid phosphatidylinositol-4,5-biphosphate (PIP_2_) using pull-down analysis [20]. The study also revealed a critical role for PIP_2_ in amphetamine-induced serotonin efflux, but not in normal serotonin transport, by SERT. Importantly, amphetamine-induced modulation of SERT transport is dependent on the formation of SERT oligomers [21]. A recent study, using super-resolution microscopy of mammalian cells expressing fluorescently tagged versions of the transporter, has provided strong evidence that PIP_2_ is critical for stability of the SERT oligomers [22]. The SERT oligomers are formed in the ER and after transport to the plasma membrane their stoichiometry is fixed [22]. Since PIP_2_ is found almost exclusively in the plasma membrane, it is suggested that the oligomeric status of the SERT is dynamic until it encounters this specific lipid in the membrane. Mutagenesis experiments suggest that two Lys residues in the cytosolic region of SERT form electrostatic interactions with the negatively charged head group of a PIP_2_ with one lipid molecule interacting with two protein molecules [22]. The suggested arrangement allows for the formation of both dimers and other higher-order oligomers. However, precise molecular details of the interaction between the lipid and SERT have yet to be elucidated.

**Figure 2.**
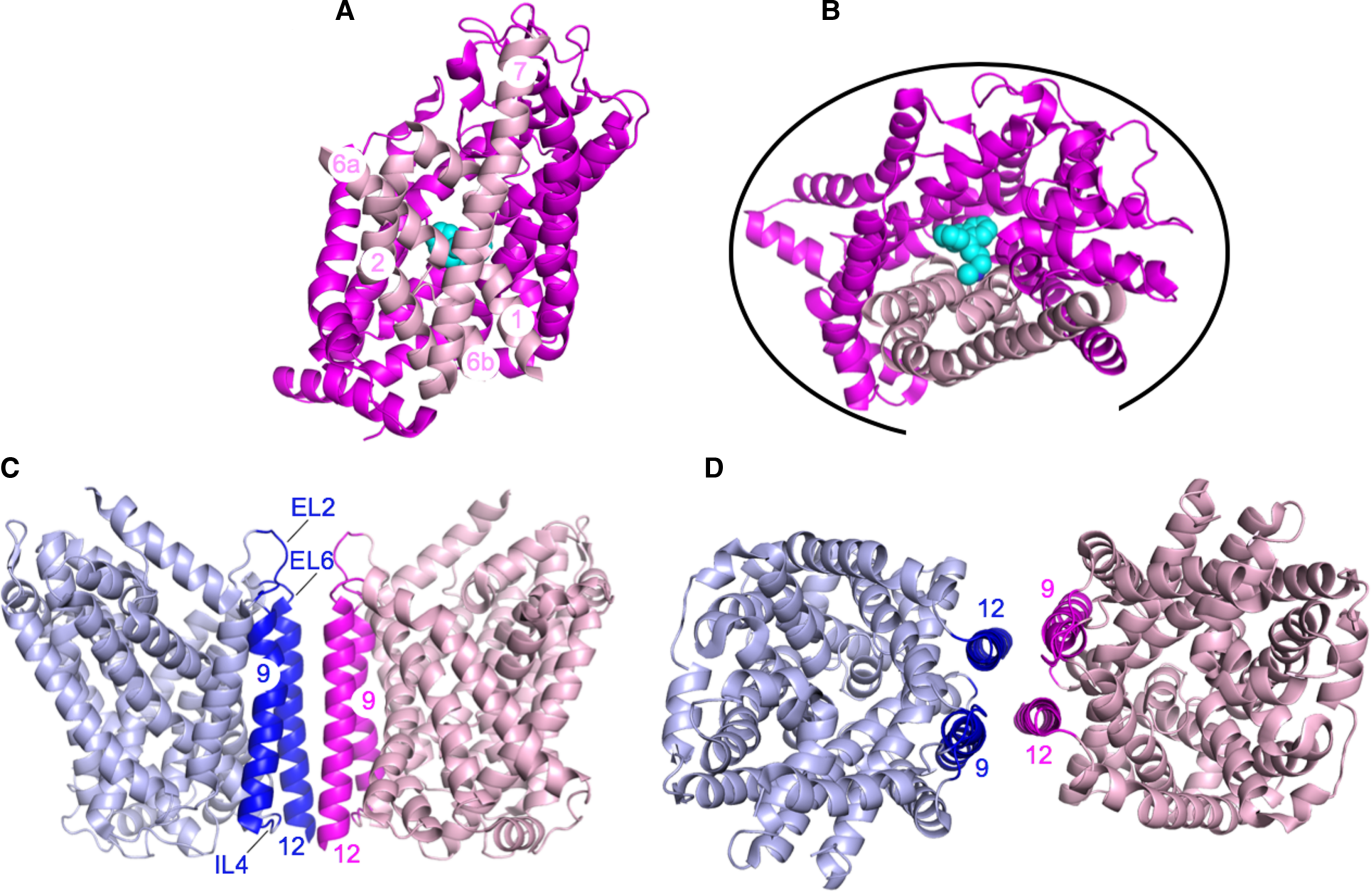
Predicted DAT dimer/oligomer interfaces. (**A**) DAT monomer (PDB: 4M48 [23]) looking through the membrane. TMHs 1, 2, 6 and 7 are coloured in pale pink with the remaining molecule coloured in magenta. The bound nortriptyline TCA, a tricyclic antidepressant, is shown in cyan. For clarity, some of the loop regions have been removed. (**B**) DAT monomer coloured and labelled as in (**A**) and shown from the extracellular side of the membrane. The black curve indicates all the regions predicted to be able form DAT:DAT interactions by MD simulations [29]. (**C**) LeuT dimer (PDB: 3TT1 [31]) looking through the membrane. One protomer is shown in pale blue and one in pale pink with the regions of the protomers (TMHs 9, 12 and extracellular loops 2 and 6 and intracellular loop 4 shown in dark blue and magenta). The different regions of the dimer interface are labelled. (**D**) LeuT dimer coloured and labelled as in (**C**) and shown from the extracellular side of the membrane.

Despite strong evidence to support oligomer formation for both SERT and the related dopamine transporter (DAT), no structures of the oligomeric forms of these proteins have been solved to date [23–27]. Recent studies have used computer modelling and molecular dynamics simulations to explore the nature of the interactions involved in oligomerisation of both proteins. Modelling, together with mutagenesis studies, indicated a DAT dimer interface involving transmembrane helices (TMHs) 6, 11 and 12 with an intermolecular disulfide bridge formed between the C306 residue at the extracellular tip of TMH6 of two protomers as a key interaction [28]. This is a little surprising given that TMH6, together with 1, 2 and 7, forms part of the so-called bundle domain responsible for transport. A more recent analysis suggested that DAT forms a range of different dimer interfaces involving virtually all the protein surface, except the vast majority of TMHs 1, 2, 6 and 7 (Figure 2A,B) [29]. The authors of this more recent study do not rule out a dimer interface involving the intermolecular disulfide bridge mentioned above; however, if a covalent interaction was involved in mediating the interactions between individual protomers, this should be captured in the crystal structure. Thus, it is possible that the disulfide bridge is an artefact of both the experimental conditions and the predicted propensity of the DAT protomers to interact in a range of different ways.

The different predicted dimer interfaces would allow the formation of higher oligomeric states [29]. The fact that there are a range of possible dimer interfaces, rather than a single defined interaction surface, means that the multiple weak interactions between individual protomers could be equally as likely to occur. This might explain the lack of crystallised DAT oligomers. A similar molecular dynamics study suggested a TMH12–TMH12 interaction interface for SERT [30]. The TMH12 is key in the formation of the dimer of LeuT, a bacterial homologue of SERT, as revealed by the crystal structure [31], although the precise interaction interface is suggested to differ between the two proteins (Figure 2C,D). Frustratingly, the lack of a high-resolution oligomeric structure for DAT and SERT continues to hamper efforts to fully understand the mechanism of action of these critical proteins.

Additional studies have probed the role of transporter oligomerisation in drug addiction. Long-term cocaine administration reduces the inhibitory effects of cocaine on the DAT, an effect associated with the addictive effects of the drug [32]. The treatment of cocaine-addicted mice with amphetamine reverses the effects of cocaine on the DAT, a process associated with inhibition of cocaine-induced DAT oligomerisation in neuroblastoma cells expressing DAT [32]. While the precise molecular regulation of DAT oligomerisation by both cocaine and amphetamine remains unclear, the oligomerisation process itself may represent a novel therapeutic target.

## Major facilitator superfamily transporters

The role of oligomerisation in transport function of the glucose transporter, GLUT1, has remained a long-standing question. It has long been known that GLUT1 forms non-covalent dimers and tetramers in the membrane [33,34]. Biochemical analysis indicates that in the tetrameric form of GLUT1, two molecules are outward facing and two inward facing at any one time, suggesting a degree of cross-talk between the individual protomers. But in the dimeric form, the individual protomers can randomly adopt inward or outward facing conformations independent of the associated protein molecule [33]. An interaction involving TMH 9 is suggested to be important for tetramer formation and high transport capacity, strongly supporting a functional role for oligomerisation [35].

**Figure 3.**
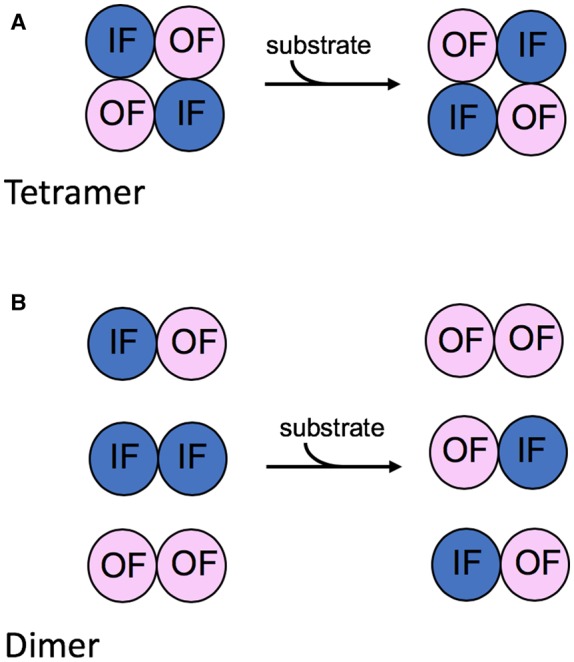
Cooperativity between the individual GLUT1 protomers. (**A**) In the tetrameric arrangement, there appears to be a concerted conformational rearrangement of the individual protomers. Substrate binding to the substrate-binding sites of two protomers exposed on either side of the membrane followed by transport results in the conformational rearrangement of all four protomers such that two protomers are always inward facing (IF) and two protomers are always outward facing (OF). (**B**) In contrast, in a dimer arrangement, the two protomers are associated but operate independently of one another.

The functional role of tetramerisation has recently been described in terms of allostery. So-called trans-allostery is the result of inhibitors binding to the endofacial (intracellular facing) binding site affecting the ability of the exofacial (extracellular facing) binding site to bind to the sugar [13]. This type of allostery is reportedly only possible in tetrameric forms of the transporter. The most plausible explanation for this is that in the tetrameric arrangement, the individual GLUT1 protomers work in pairs (Figure 3). One protomer in a pair is inward facing, while the other must be outward facing. Sugar binding to one site and subsequent transport causes a conformational rearrangement of that transport module which is linked to the opposing rearrangement in the associated molecule [13]. The tetrameric arrangement of the molecules may be necessary to provide sufficient structural stability to allow these concerted conformational changes. It certainly seems possible that such an alternating inward:outward arrangement of the protomers occurs in other transporters such as UapA, and this would certainly account for the dominant negative phenotypes observed for some UapA mutants [8].

The structural basis of the intermolecular interactions within the GLUT1 tetramer must allow for this type of transporter cross-talk. Intriguingly, however, rather like the situation with neurotransmitter transporters described above, these oligomeric states have not been captured in the recent high-resolution X-ray crystallographic structures of human GLUT1 [36], human GLUT3 [37] and the related bovine and rat GLUT5 [38]. TM9 is involved in crystal packing in crystals of human GLUT3, although in a non-physiological linear arrangement of transporter molecules [37]. It is surprising that the oligomeric states of the proteins have not been successfully crystallised given that early studies demonstrated the presence of tetramers and dimers in cholate-containing solution [33,39]. This difference in the oligomeric status in the crystal structures may reflect the different conditions used for isolation and crystallisation; dodecyl-β-d-maltoside was used for isolation of all three GLUT transporters, with GLUT1 being crystallised in nonyl-β-d-glucoside and GLUT3 being exchanged into 6-cyclohexyl-1-hexyl-β-d-maltoside (Cymal-6) prior to crystallisation in lipidic cubic phase. Alternatively, the early studies had used the native erythrocytes as a source of material, while for the more recent structural studies the proteins were expressed heterologously in insect cells (GLUT1, GLUT3) and yeast (GLUT5). Differences in lipid composition or other key factors in these heterologous systems may preclude the formation of oligomers stable enough to withstand the extraction and isolation process.

## Sodium bile acid co-transporter family

The human apical sodium-dependent bile acid transporter (hASBT) is responsible for uptake of bile acids from the ileum and has roles in cholesterol homeostasis [40,41]. There is some evidence to indicate that hASBT, and other SLC10A family members, exists as dimers and possibly higher-order oligomers [42], although the molecular basis of oligomer formation and the role of oligomerisation in transporter function were unknown. Studies with a Cys-less version of hASBT indicated that oligomerisation was independent of disulfide bridge formation [43]. Importantly, data obtained from this study also demonstrated that the functionally inactive Cys-less mutant exerts a dominant negative effect on transport activity of co-expressed WT transporter. These findings strongly suggest that the oligomer is the functional unit and cross-talk between the individual protomers is critical for transport activity [43]. High-resolution structures of bacterial homologues of the hABST [44,45] revealed only monomeric forms of the proteins. The absence of oligomers for the bacterial proteins may be a result of the isolation and crystallisation process and also may reflect differences in the organisation of the transporters in prokaryotes versus higher eukaryotes.

## Conclusions

In the two short years since we wrote our first review on the functional and structural implications of transporter oligomerisation [46], the field has moved on remarkably. Of particular importance have been the novel insights into the roles of lipids in transporter oligomerisation and function. New models of functional interdependency between individual oligomeric transport protomers are emerging, and it is likely that these will be relevant to a range of transport mechanisms. Additional recent oligomeric transporter structures include EIIC sugar transporter family members [47,48] and the human neutral amino acid transporter ASCT2 [49]. Although there is no evidence as yet that these are functionally relevant this illustrates that there are many systems yet to be fully studied in terms of the role of oligomerisation. Perhaps, most excitingly, the first hints have emerged that transporter oligomerisation itself might represent a drug target.

## Abbreviations

AE1: anion exchanger 1
DAT: dopamine transporter
hASBT: human apical sodium-dependent bile acid transporter
MS: mass spectrometry
PE: phosphatidylethanolamine
PI: phosphatidylinositol
PIP_2_: phosphatidylinositol-4,5-biphosphate
SERT: serotonin transporter
TMHs: transmembrane helices
